# The Timing of IFNβ Production Affects Early Innate Responses to *Listeria monocytogenes* and Determines the Overall Outcome of Lethal Infection

**DOI:** 10.1371/journal.pone.0043455

**Published:** 2012-08-17

**Authors:** Francesca Pontiroli, Olivier Dussurget, Ivan Zanoni, Matteo Urbano, Ottavio Beretta, Francesca Granucci, Paola Ricciardi-Castagnoli, Pascale Cossart, Maria Foti

**Affiliations:** 1 Department of Biotechnology and Bioscience, University of Milano-Bicocca, Milan, Italy; 2 Genopolis Consortium, University of Milano-Bicocca, Milan, Italy; 3 Unité des Interactions Bactéries-Cellules, Institut Pasteur, Paris, France; 4 Inserm U604, Paris, France; 5 INRA USC2020, Paris, France; 6 Singapore Immunology Network, Singapore, Singapore; Institut de Pharmacologie et de Biologie Structurale, France

## Abstract

Dendritic cells (DCs) and natural killer (NK) cells are essential components of the innate immunity and play a crucial role in the first phase of host defense against infections and tumors. *Listeria monocytogenes* (*Lm*) is an intracellular pathogen that colonizes the cytosol of eukaryotic cells. Recent findings have shown *Lm* specifically in splenic CD8a^+^ DCs shortly after intravenous infection. We examined gene expression profiles of mouse DCs exposed to *Lm* to elucidate the molecular mechanisms underlying DCs interaction with *Lm*. Using a functional genomics approach, we found that *Lm* infection induced a cluster of late response genes including type I IFNs and interferon responsive genes (IRGs) in DCs. Type I INFs were produced at the maximal level only at 24 h post infection indicating that the regulation of IFNs in the context of Lm infection is delayed compared to the rapid response observed with viral pathogens. We showed that during *Lm* infection, IFNγ production and cytotoxic activity were severely impaired in NK cells compared to *E. coli* infection. These defects were restored by providing an exogenous source of IFNβ during the initial phase of bacterial challenge. Moreover, when treated with IFNβ during early infection, NK cells were able to reduce bacterial titer in the spleen and significantly improve survival of infected mice. These findings show that the timing of IFNβ production is fundamental to the efficient control of the bacterium during the early innate phase of *Lm* infection.

## Introduction

Protective immunity requires the coordinated activation of both the innate and adaptive immune systems. Interactions between innate and adaptive immune effectors are essential for the efficient control of pathogens and tumors and often play an important role in ending immune responses which would otherwise eventually be harmful to the host. DCs and NK cells play an essential role in the first phase of pathogen infection. Mice infected with the intracellular Gram-positive bacterium *Listeria monocytogenes* (*Lm*) are often used as a model to study innate and adaptive immunity [1], [2]. Major sites of infection with *Lm* are the spleen and liver, where bacteria are found preferentially within the cytosol of antigen-presenting cells (APC) and hepatocytes. *Lm* can spread from cell to cell without leaving the intracellular compartment, which is the main reason why *Lm*-specific CD8+ T cells are needed for bacterial clearance and for providing protective immunity. In the absence of T cells, chronically persistent infections can develop [3]. Bacterial growth during the first days after infection is mainly controlled by cells belonging to the innate immune system, including neutrophils, NK cells, and macrophages [4], [5]. Upon infection in the murine spleen, *Lm* is first found within macrophages and DCs in the marginal zone between the T cell-rich white pulp and the B cell-rich red pulp [5], [6], [7]. These infected cells then migrate to the white pulp region and form the beginning of a focal infection that expands as neighboring cells become infected by the intercellular spread of bacteria. The cytokine IFNγ plays a major role in the control of *Lm* infection during both the innate and adaptive immune responses. NK cells and NKT cells secrete IFNγ and are thought to limit exponential growth of the bacteria primarily by activating macrophages during the first few days of infection [8], [9]. IFNγ secretion by these cells is also thought to promote a Th1-type response against *Lm* by increasing MHC class I expression.

In addition to ‘bridging’ innate and adaptive immunity, DCs may also contribute to primary resistance against infection. Recent reports have suggested that DCs themselves may be involved in innate defense against infections [10]. In *Lm* infection, previous findings have suggested that a CD11c^+^ DCs subset characterized by the production of tumor necrosis factor alpha (TNF-a) and inducible nitric oxide synthase (iNOS) is essential for early control of bacterial growth [11].

In addition to the major role of DCs during T-cell priming, a recent study reported an important role for DCs in CD8^+^ memory T-cell responses upon secondary infection with *Lm* [12], [13]. Localization of *Lm* to the cytosol is required for virulence and for recognition of the bacterium by intracellular DNA sensing machinery. *Lm* produces a hemolysin, listeriolysin O (LLO), which permits the bacterium to destroy phagosomes and escape into the cytosol of infected cells. Consequently, strains lacking expression of LLO (DHly) are avirulent. In addition, *Lm* DHly fails to elicit the production of IFN-αβ by infected macrophages [14]. Production of IFN-αβ during *Lm* infection is thought to be dependent on the detection of microbial products by a receptor present in the host cell cytosol [15]. Nevertheless, IFN-β induction by a TLR2-dependent mechanism has been also reported [16]. The DCs cytosol is a major host–pathogen interface during *Lm* infection. We analyzed events during host-pathogen interactions, by comparing the DCs response to two *Listeria* strains, *Lm* and the non pathogenic *Listeria innocua* (*Li*). *Lm* localizes to the cytosol whereas *Li* does not. Entry into the DCs cytosol activates a complex gene reprogramming that results in the release of proinflammatory cytokines, type I IFNs and chemokines. We describe a *Lm*-specific delay of type I IFN induction compared to *E.coli* in DCs and show that this delay has marked effects on NK cell activation and on mouse survival to lethal Lm infections. Thus, *Lm* may evade innate immune surveillance specifically by disruption of the DCs-NK cell interaction and by modification on other regulatory mechanisms through an improper induction of type I IFNs.

## Results

### *In vitro* Production of Type I IFNs by DCs Infected with *Listeria Monocytogenes* is Delayed Compared to DCs Infected with other Bacteria

We studied the host responses to Gram-positive bacteria of the genus *Listeria,* specifically, the pathogenic species *Listeria monocytogenes* (*Lm*) and the non-pathogenic species *Listeria innocua (Li)*. Genome organization is conserved among many *Listeria* species and they frequently have a high number of genes in common. However, some *Listeria* species, such as *Lm*, carry specific genes that allow them to cause disease in humans and animals [17]. We investigated the interactions between these two *Listeria* strains and DCs using microarray technology. We compared the transcriptomes of *Lm*- and *Li*-infected D1 cells, a spleen-derived murine DC line [18], at 2, 4, 8, 12 and 24 hours post-infection (p.i.). The D1 cells were exposed to *Lm* or *Li* at a multiplicity of infection (MOI) that was optimal for triggering the up-regulation of co-stimulatory molecules. Examples of flow cytometry profiles of D1 cells infected with *Lm, Li* and *E. coli* are shown in Figure S1. After normalization and filtering for gene quality control in the microarray analyses, 10,347 probe sets remained for further evaluation. We selected only those genes for which the differential expression changes were 2.0-fold or greater and were statistically significant (one-way ANOVA, p<0.0001). We found 1,775 genes in the *Lm*-infected group and 2,219 genes in the *Li*-infected group which had at least a 2.0-fold change in transcription level relative to those in the uninfected control cells. These differentially expressed genes were studied in further detail in an effort to understand the mechanisms of the host response to these Gram-positive bacteria.

**Figure 1:**
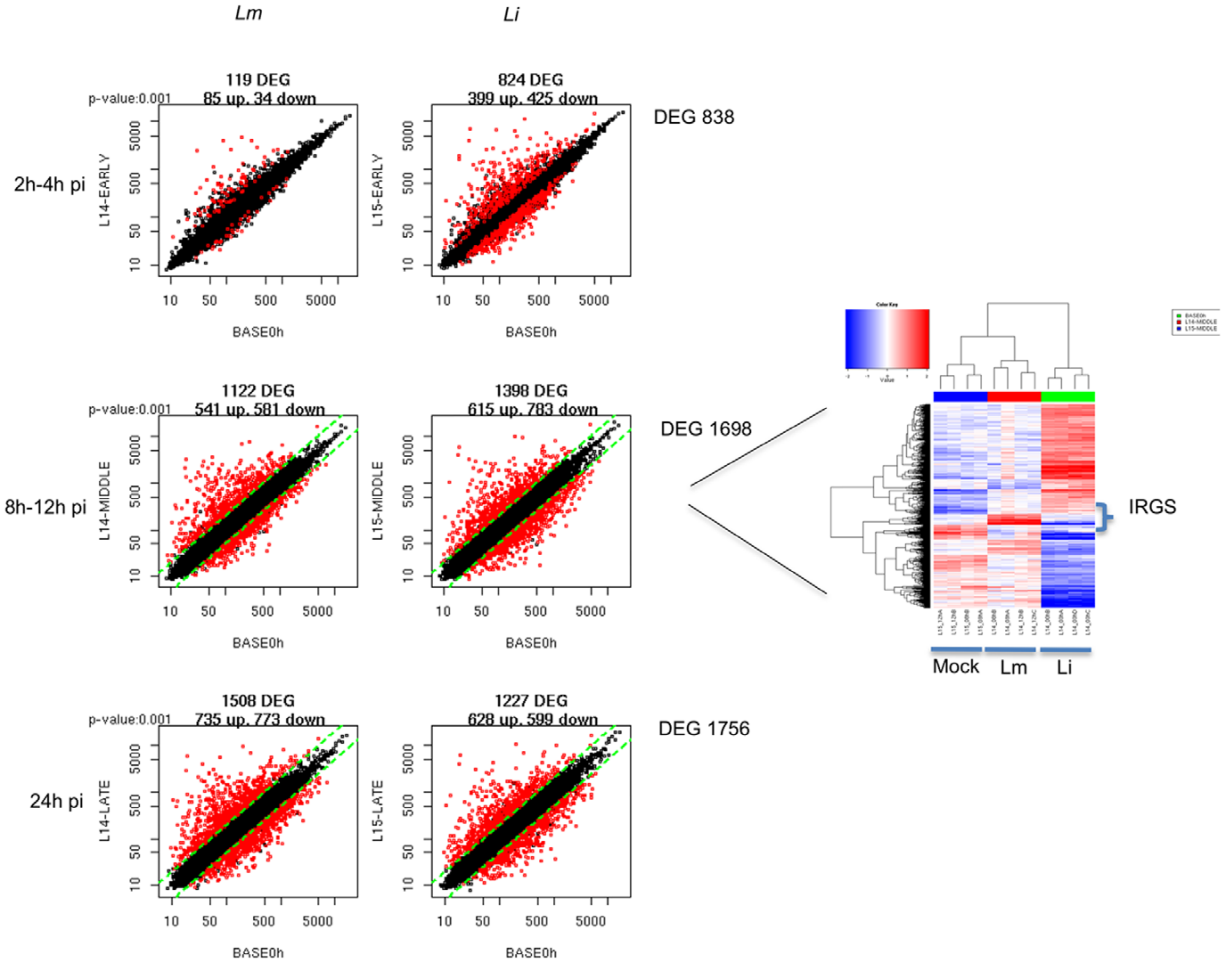
Differentially expressed genes induced by *Listeria* strains in DCs. Visualization by scatter plot analysis of the differentially expressed genes (DEGs) induced in D1 cells by *L. monocytogenes* (Lm) and *L. innocua* (Li) infections. The MOI of infections were 1∶40 for Lm and 1∶1000 for Li. Scatter plot analyses of early (2–4 hr p.i.), middle (8–12 hr p.i.) and late (24 hr p.i.) modulated genes are shown. Signal log ratios of genes expressed by D1 cells at different time points after bacteria exposure were compared to the untreated samples. Red dots represent genes showing a fold-change of 2 or more with respect to untreated samples. The middle response (8–12 hr) is also represented as a heat map to specifically show the presence of interferon-responsive genes (IRGs) differentially regulated by the two bacteria.

Consistent with previous studies of macrophages [19], we found that the transcriptional responses could be broadly divided into “early" (2−4 hr p.i.), “middle" (8−12 hr p.i.) and “late" (24 hr p.i.) responses. At early time points, *Li* infection induced a stronger transcriptional response (824 genes) than that induced by *Lm* infection (119 genes). Only at 24 hr was the response to *Lm* infection (1,508) higher than the response to *Li* infection (1,227) (Figure 1). An unsupervised analysis revealed groups of genes with similar changes in expression. A large number of genes encoding receptors, signaling molecules and transcription factors, as well as genes encoding adhesion molecules, enzymes and anti-apoptotic molecules and genes involved in tissue remodeling were differentially regulated in DCs infected with *Listeria*. Particularly, gene clusters corresponding to the early response were enriched in genes involved in the “*cytokine-cytokine receptor interaction"* (KEGG pathway number 04060). The response of *Li*-infected DCs (29 genes; p = 9.15×10^−9^) was more highly enriched in these genes than was the response of *Lm*-infected DCs (14 genes; p = 1.92×10^−7^) (Figure S2). GO functional term annotation revealed different enrichment profiles for the two different *Listeria*-infected DCs, which reflected the different numbers of genes induced at the early time points. The biological processes most enriched in the *Lm*-infected cells were “*locomotion*" (GO0040011; p = 2.63×10^−10^) and “*chemotaxis*" (GO0006935; p = 1.53×10^−9^). In contrast, “i*mmune system process*" (GO0002376; p = 5.59×10^−9^) and “*immune response*" (GO0006955; p = of 6.03×10^−8^) were the biological processes most enriched in the *Li*-infected cells (Figure S3). The data revealed that at early times (2−4 hr p.i.), *Li* bacteria induced a greater transcriptional response in DCs, which was characterized by a broad induction of immune system genes. In contrast, the functional classes of genes that were most enriched among those induced by *Lm* bacteria were specifically related to chemotaxis and locomotion.

**Figure 2:**
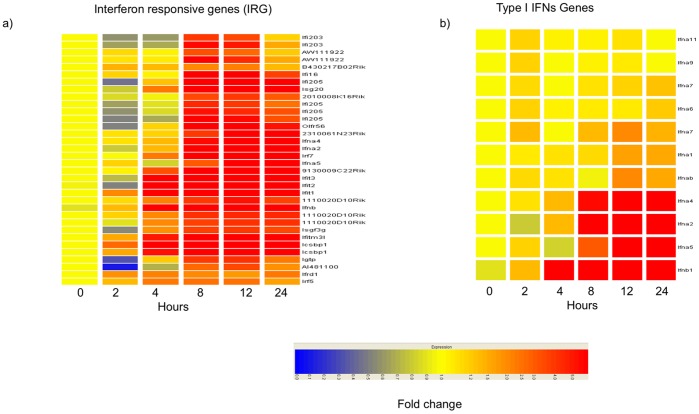
Induction of type I IFN genes. Samples derived from two biological replicates per time point were used for subsequent GeneChip probe arrays. (A) Heat map of the time course of IRG expression in DCs after exposure to *Lm*. (B) Heat map of the time courses of IFNβ and IFNα gene expression. RNAs were collected at different time points and the gene expression levels were measured by microarray analysis. Heat maps were generated with the GeneSpring hierarchical clustering algorithm. Data show mean fold-changes normalized to the pre-exposure 0 hr time point.

Next, we compiled a list of all the genes induced by the two bacteria and identified those that were induced by both species (1,361) and those that were specifically induced by *Lm* (414) or by *Li* (858). Functional annotation of the genes specifically induced by *Li* revealed statistically significant enrichments for “*phosphate metabolic process*", “*regulation of apoptosis*" and the “*MAP kinase signaling pathway*" (Figure S4 and data not shown). The genes induced specifically by *Lm* were mainly part of the “*response to virus*" class (Oas1b, ISG15, Tgtp2, Ifna2, Ifna4, Ifna5, Ifnb, Mx2 and POLR3C). In particular, the induction of these genes was most evident in the middle time points (8 hr and 12 hr p.i.) and included a large number of genes induced by type I IFN signaling. This finding suggests that the detection of *Lm* in the host cytosol is associated with a type I IFN response in DCs, as has been reported previously for macrophages and other cells [14], [20], [21]. The late cluster of the *Lm*-infected DCs was characterized by the presence of interferon-regulated genes (IRG) (Figure 2A) and by different type I IFN subtype genes (Figure 2B). Changes in IFNβ gene transcription were first observed at 4 hr p.i., whereas an effect on IFNα genes was first detected at 8 hr p.i. (Figure 2B).

**Figure 3:**
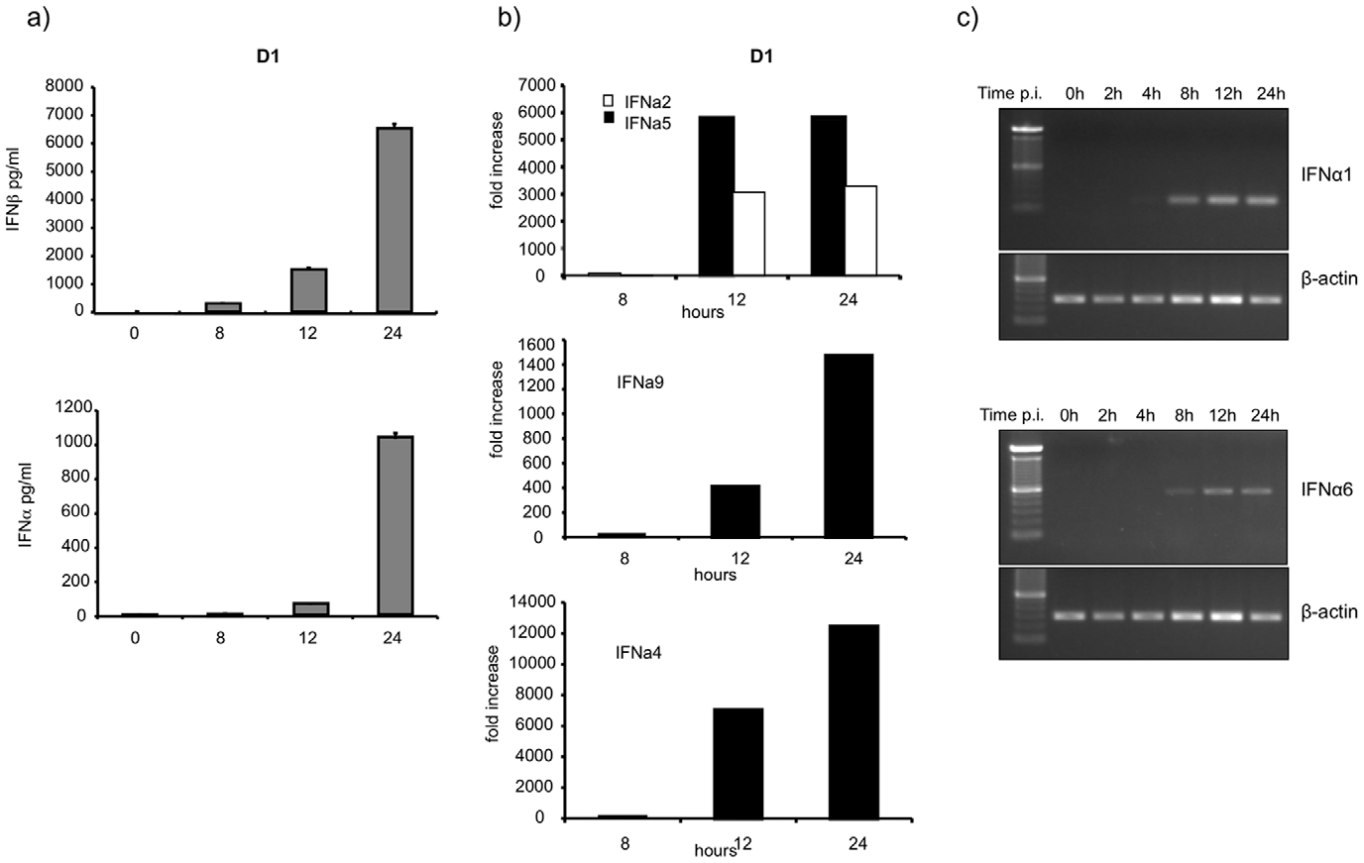
Type I IFN production by *Lm*-infected DCs. (A) D1 cells were infected with *Lm* at an MOI of 40. Supernatants were collected at different time points and IFNβ and IFNα levels were quantified by ELISA. (B) D1 cells were infected with *Lm* at an MOI of 40. RNA was extracted at different time points and used for qRT-PCR analysis. The fold-increases, relative to β-actin, for IFNα4, IFNα9, IFNα5, IFNα2 are shown. (C) D1 cells were infected with *Lm*, and the RNA was extracted and analyzed by RT-PCR. IFNα6 and IFNα1 mRNA levels are shown. Data shown are representative of at least three independent experiments.

To confirm data obtained from the transcriptional analysis, we measured IFNβ and IFNα production levels in infected D1 cells. Variable levels of IFNα and IFNβ proteins were released during *Lm* infection, and peak production occurred at 24 hr p.i. (Figure 3A). Because the anti-IFNα antibody used in the ELISA was unable to distinguish between different IFNα subtypes, we used quantitative real-time reverse transcription PCR (qRT-PCR) to analyze the expression of IFNα4, IFNα9, IFNα2 and IFNα5, and we used an RT-PCR analysis to detect IFNα1 and IFNα6. We were unable to analyze IFNα7, IFNα11, IFNα12 and IFNα13 expression due to the absence of oligonucleotide sequence specificity. Transcripts for IFNα4, IFNα2, IFNα5 and IFNα9 were all induced at 12 hr and 24 hr p.i. The highest level produced was for IFNα4 (12,000 times the IFNα4 transcript level in untreated controls) and the lowest level of induction was for IFNα9 (1,400 times the level in the untreated controls) (Figure 3B). IFNα1 and IFNα6 gene induction were also detected in *Lm* infections in DCs at 8 hr, 12 hr and 24 hr p.i. (Figure 3C).

### Type I IFNs are Induced in Bone-marrow Derived DCs (BMDCs)

We then investigated the ability of *Lm* infection to induce type I IFNs in freshly isolated BMDCs. Although type I IFNs are known to be induced in response to *Lm,* we were interested in understanding whether delayed production of these important cytokines could affect the generation of very early innate responses. We therefore compared the host response production of type I IFNs using bacteria that modulate these genes through different cellular pathways (cell membrane stimulation versus intracellular stimulation), which result in different type I IFN production kinetics.

**Figure 4:**
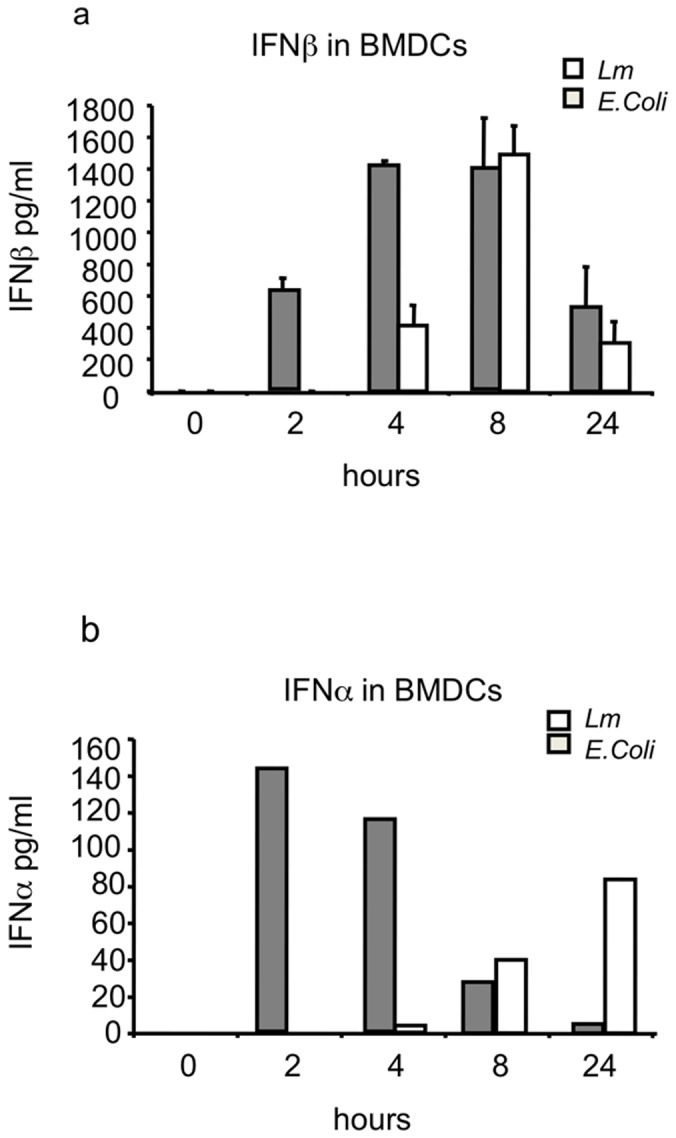
Type I IFN production by BMDCs. BMDCs from C57BL/6 mice were activated with *Lm* or *E. coli* at an MOI of 20. (A) IFNβ production by BMDCs. (B) IFNα production at the time points indicated was evaluated by ELISA. Data shown are representative of at least three independent experiments.

We infected wild-type BMDCs with *Lm* or the Gram-negative bacterium *E. coli* at an MOI of 20 and measured type I IFN production in the supernatant at different time points. As *E. coli* LPS signals through the cell surface Toll-like receptor (TLR) TLR4, *E. coli* is known to induce early production of type I IFNs [22]. As expected, type I IFN production was efficiently induced in BMDCs soon after *E. coli* infection. *Lm* infection also induced type I IFN production in BMDCs, but as this requires cytosolic detection by these cells, the kinetics of IFN secretion differed substantially from those triggered by *E. coli*. During *Lm* infection, IFNβ secretion was first detected at 8 hr p.i., whereas, in *E. coli*-infected cells, the main peak of IFNβ production occurred as early as 4 hr p.i. (Figure 4A). The difference was even more evident for IFNα: whereas *E. coli* induced a peak in IFNα production at 2 hr p.i., induction by *Lm* did not begin until 8 hr p.i., reaching the maximum level at 24 hr p.i. (Figure 4B). Thus, *Lm* induced a late, type I IFN response in D1 cells and in BMDCs, which was consistent with our microarray data and predicted by the detection system activated by this microorganism in DCs.

### *Lm* Infection *in vivo* Induces a Late IFNβ Response in the Spleens of Infected Mice

**Figure 5:**
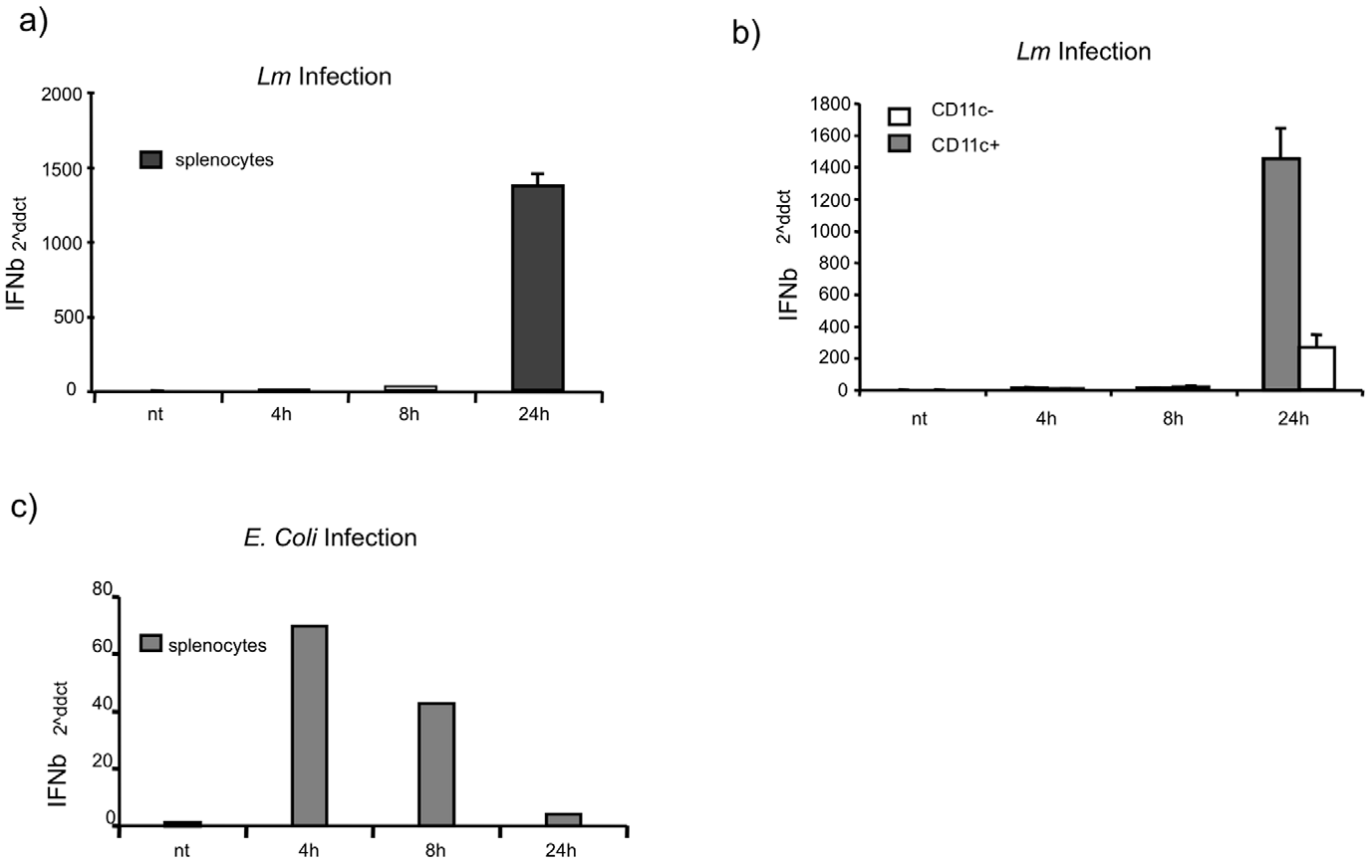
*In vivo* IFNβ production during *Lm* infection. Mice (n = 5/group) were injected with 1×10^6^ CFU of *Lm* and *E. coli*. RNA was extracted at 4 hr, 8 hr and 24 hr p.i. and the IFNβ mRNA was quantified. (A) IFNβ gene expression from total spleen at the time points indicated. (B) IFNβ gene expression in CD11c^+^ and CD11c^−^ cells purified from total spleen. (C) IFNβ gene expression in total spleen from mice infected with *E. coli* (1×10^6^ CFU) as a positive control. The housekeeping gene *PPIA* was used as a reference to normalize data. Data shown are representative of at least three independent experiments.

The finding that cytosolic *Lm* detection is responsible for the late production of type I IFNs in DCs compared to the early IFN production caused by cell surface receptor triggering, prompt us to verify whether these different type I IFNs kinetics are also measurable *in vivo* when mice are challenged with live bacteria. We focused on the production of IFNβ as it appears to be the primary type I IFN family member that is induced after infection with *Lm*. We infected C57BL/6 mice with *Lm* or *E. coli* (10^6^ CFU) and extracted total RNA at different time points from whole spleens and from CD11c^+^ cells purified by magnetic bead cell sorting. The sorted CD11c^+^ cells were enriched for IFNβ production in response to *Lm*, whereas the CD11c^−^ cells showed a much weaker response. IFNβ mRNA was detectable in total spleen lysate (Figure 5A) and in CD11c^+^ cells of *Lm*-infected mice (Figure 5B) at only 24 hr p.i. In contrast, *in vivo* infection with *E. coli* induced IFNβ production at an earlier stage as IFNβ gene induction occurred at 4 hr and 8 hr p.i. (Figure 5C). CD11c^+^ DCs were involved in the production of this cytokine in the spleen of *Lm*-infected mice. Thus, these data confirm that IFNβ production is strongly induced in the CD11c^+^ fraction of spleen cells and that this production is delayed in the mouse model of systemic *Lm* infection relative to the timing of the IFNβ produced in response to *E. coli* infection.

### IFNβ Treatment during the First Few Hours after *Lm* Infection Improves the Survival Outcome of Lethally Infected Mice

**Figure 6:**
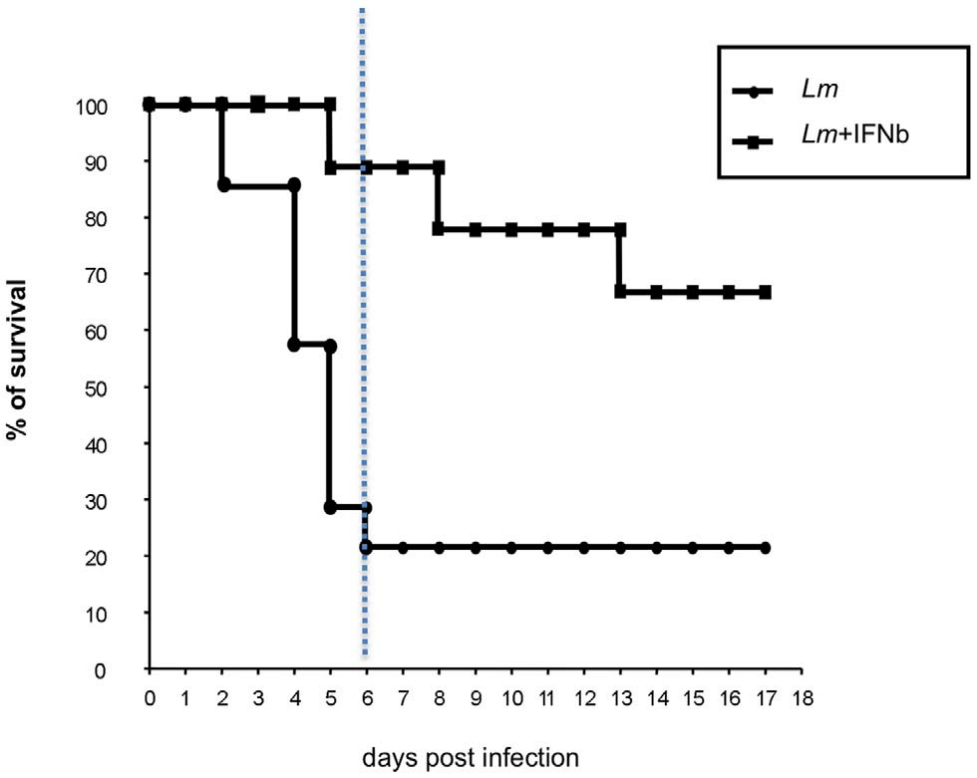
Survival assay of mice infected with *Lm*. Mice (n = 5/group) were injected with a lethal dose of *Lm* alone (1×10^6^ CFU) or with IFNβ (38,000 U). Survival was monitored daily, for up to 18 days. Data shown are representative of at least three independent experiments. All experiments were performed using protocols approved by University of Milano-Bicocca Animal Care and Use Committee. Mice were housed in containment facilities of the animal facility and maintained on a regular 12∶12 hour light:dark cycle with food and water ad libitum.

We performed a survival assay to determine whether the presence of an early source of IFNβ could affect the survival of mice injected with a lethal dose of *Lm*. We injected mice intravenously with *Lm* (10^6^ CFU), with or without a subsequent injection of IFNβ, and then their survival was monitored. Early IFNβ treatment significantly prolonged the survival of mice injected with a lethal dose of *Lm* by up to one week (Figure 6). Mice injected with *Lm* alone had a low survival rate after one week (20%), whereas mice injected with *Lm* and IFNβ had a survival rate of 90% at this same time point. These results indicate that without the presence of IFNβ during the early phase of *Lm* infection, mice were unable to mount a rapid innate immune response *in vivo,* resulting in poorer control of bacterial spread. Thus, in our experimental model, the timing of IFNβ production is crucial for controlling Lm lethal infection.

We speculate that *Lm* does not activate the full innate immune response during the very early phase of infection. As treatment of mice with IFNβ increased their survival after infection with a lethal dose of bacteria, IFNs appear to triggered antibacterial activity *in vivo* and may enhance innate immunity during intracellular microbial infections.

### BMDCs Infected with *Lm* are Severely Impaired in their Capacity to Elicit IFNγ Production by NK Cells

The observation that delayed induction of type I IFN affected the strength and the quality of the early innate immune response prompted us to study the interaction between *Lm*-infected DCs and NK cells. Type I IFNs have been primarily associated with antiviral responses and their role in antibacterial immunity has remained unclear [23], [24]. Type I IFNs are produced immediately after pathogen infection and profoundly affect the nature of innate and adaptive immune responses [25]. Therefore, we investigated whether the late induction of type I IFN genes has a functional effect on the innate immune response to *Lm*. Recovery from infection depends on the host’s ability to mount effective early innate responses to control the invading pathogens. During Gram-negative bacterial infections, early type I IFN production by DCs (during the first five hours) is essential for the initial induction of antibacterial NK cell function [22]. Activation of NK cells during the innate phase of the immune response has also been shown to be important in controlling the spread of a variety of microbial pathogens [26]. We therefore hypothesized that a delay in type I IFN production by DCs following *Lm* infection may cause a delay in NK cell activation, markedly affecting the development of an efficient innate response able to control the infection.

**Figure 7:**
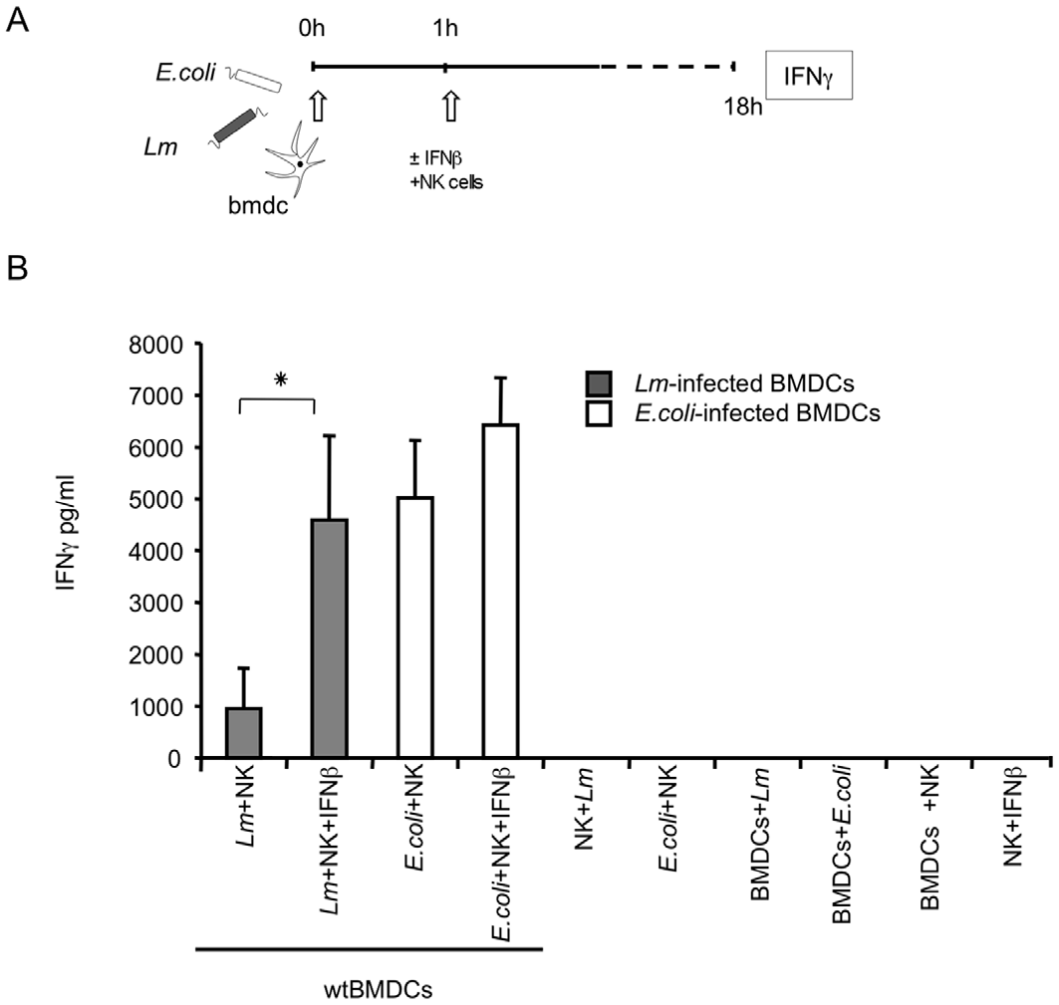
IFNγ production by NK cells cultured with BMDCs stimulated with *Lm* or *E. coli*. BMDCs were infected with *Lm* or *E. coli* at an MOI of 20 and cultured with syngeneic NK cells for 18 hr. Where indicated, recombinant IFNβ was added to the co-culture one hour after infection. Levels of IFNγ in the culture supernatants were quantified by ELISA. (A) Experimental design. (B) Co-culture experiments. The means ± SDs of three independent experiments are shown. p value <0.01.

We first studied the ability of *Lm*-infected BMDCs to elicit IFNγ production by syngeneic NK cells. BMDCs were activated with *Lm* or *E. coli* at a MOI of 20. Syngeneic NK cells were then added to the co-culture after one hour. At 18 hr, culture supernatants were collected and the levels of IFNγ in the supernatants were measured by ELISA. Whereas *E. coli*-infected BMDCs induced IFNγ production by syngeneic NK cells as previously described [22], the capacity of *Lm-*infected BMDCs to induce IFNγ production was severely impaired (Figure 7). Induction of IFNγ production in NK cells was fully restored by adding exogenous recombinant IFNβ to the BMDC-NK cell co-cultures immediately after activating the BMDCs with *Lm* (Figure 7).

These data confirm our hypothesis that the inability of *Lm*-infected DCs to produce type I IFNs during the early stages of bacterial infection, at least *in vitro*, disrupts the activation of NK cells.

### NK Cell Activation is Enhanced in Response to IFNβ Treatment *in vivo*

**Figure 8:**
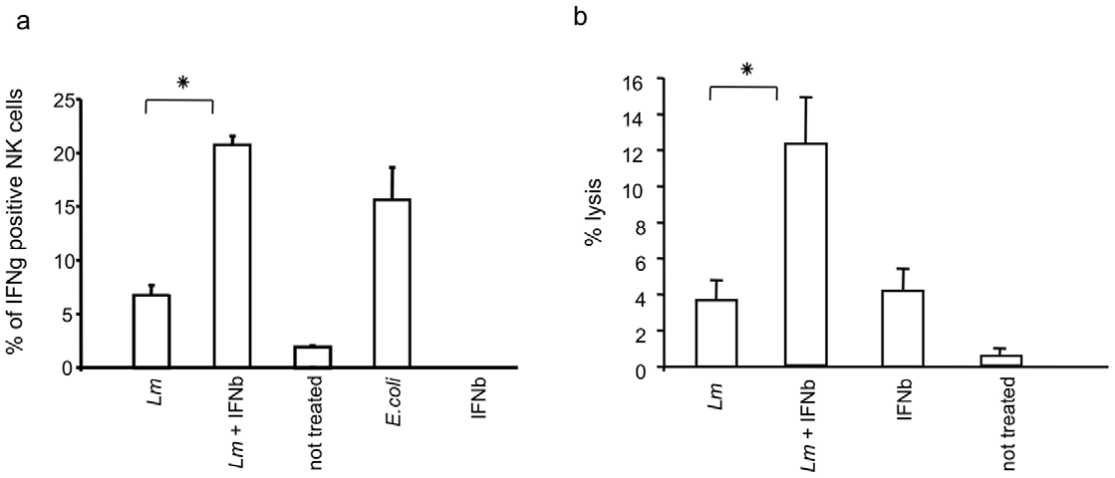
NK cell activation in the spleen of mice infected with *Lm*. Mice (n = 5/group) were infected with *Lm* (1×10^6^ CFU)± IFNβ. Spleens were removed five hours after infection and NK cell activation was evaluated. (A) Intracellular staining for IFNγ in DX5-positive cells. (B) Splenocytes were co-cultured with YAC-1 cells and the percentage of target cell lysis was determined after three hours; p-value <0.01. The mean of three independent experiments is shown.

Given that an early source of IFNβ was required *in vitro* to elicit IFNγ production by NK cells and that, during *in vivo Lm* challenge, induced IFNβ production was maximal at 24h p.i., we investigated whether delayed IFNβ production also affects NK cell activation *in vivo*. We infected mice with 10^6^ CFU of *E. coli*, as an early IFNβ inducer, or with 10^6^ CFU of *Lm* either with or without subsequent injection of rIFNβ (38,000 U/mouse). IFNγ production in the NK cells of infected mice was assessed after five hours of infection by staining for intracellular IFNγ in DX5^+^ cells. Consistent with our *in vitro* data, only a small percentage of NK cells (6%) in *Lm*-infected mice were positive for IFNγ production (Figure 8A). Thus, mice infected with *Lm* were unable to mount a full NK cell response, in terms of IFNγ production, during the very early stages of infection. Injection with rIFNβ one hour after bacterial challenge resulted in a substantially higher percentage of IFNγ-positive NK cells (22%) in *Lm*-infected mice, which was similar to the percentage found in *E. coli*-infected mice (Figure 8A).

**Figure 9:**
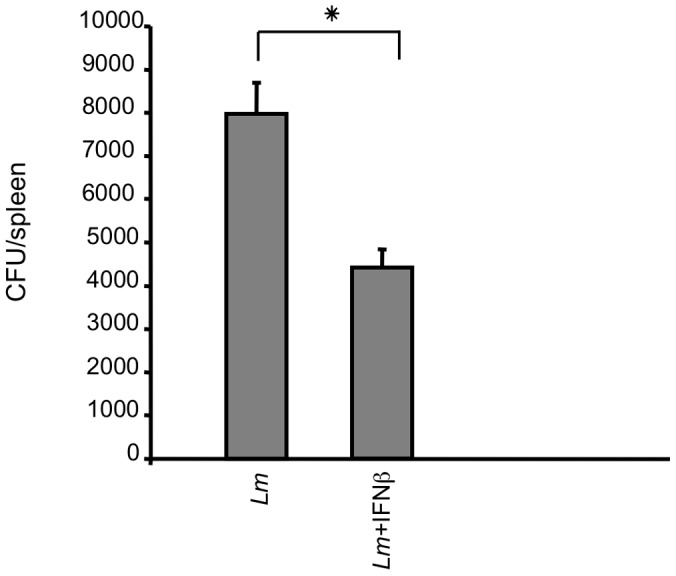
Bacterial burden in the spleen of mice infected with *Lm*. Bacterial burden was measured in five mice per group. Mice were infected with sub-lethal doses (5×10^4^ CFU) of *Lm* ± IFNβ and killed after five hours. *Lm* titers were determined in the spleen and expressed as CFUs. Recombinant IFNβ was given one hour post-infection, as indicated; p = 0.0001. The mean of three independent experiments is shown.

We then measured the cytotoxic activity of NK cells from the spleens of mice treated with *Lm* and rIFNβ, with *Lm* only or with rIFNβ only. The capacity of NK cells to kill YAC-1 cell targets was higher (15% lysis) in mice injected with rIFNβ one hour after bacterial challenge than in mice injected with *Lm* (4% lysis) alone or rIFNβ (5% lysis) alone (Figure 8B). To assess whether exogenous rIFNβ was associated with improved control of bacterial growth, the bacterial burdens were evaluated in the spleens of *Lm*-infected mice at 5 hr p.i. At this early time point, the mean bacterial titer in the spleens of *Lm*-infected mice treated with rIFNβ was moderately but significantly lower than the mean titer in the spleens of *Lm*-infected mice lacking rIFNβ treatment (Figure 9). These results suggest that the protective action of rIFNβ is only partially due to the reduction of bacterial growth, which implies that additional mechanisms are primarily responsible for the effects of IFNβ treatment. Type I IFNs mediate many anti-cellular effects by modulating cell viability and function which ultimately may lead to the induction of disease tolerance instead of disease resistance [27]. In conclusion, our results suggest that the production or presence of early IFNβ is important for regulating innate responses during the initial phase of *Lm* infection.

## Discussion

The analysis of bacteria-host cell interactions at the molecular and cellular levels has become a major research area in recent years [28], [29]. Host defenses against intracellular pathogens such *Lm* require the coordinated interactions of a number of innate and adaptive immune system components to clear the infection [1], [30], [31], [32]. Early activation of innate immune cells is important for host survival and bacterial clearance [4], [6], [11], [33], [34], [35], [36] whereas development of adaptive immunity is crucial for long-term protection and for providing sterilizing immunity. However, it is becoming clear that in the context of *Lm* challenge, innate immunity alone is unable to completely control the infection and the development of adaptive immunity is necessary to fully eliminate the pathogen.

The general requirement of DCs during *Lm* infection has been studied by using different experimental models but the exact role of DCs in combating infection remains unclear [5], [13], [37], [38], [39], [40]. A subpopulation of CD11c^+^ cells has been implicated in various stages of *Lm* immunity, including splenic sequestration [5], [41], [42], early activation of CD8^+^ T-cells [43].

Expression profiles have been widely used to determine the molecules involved in complex host-pathogen interactions [44].

Post-infection gene expression has been evaluated at short/middle time points (at 0 hr and at 1−8 hr p.i.) [15], [19], [45] and with end-point assays [45]. Nevertheless, no previous study has addressed the genome-wide DC-specific response during the first 24 hours of *Lm* infection. Microarray analyses have shown that the IFNβ gene and interferon-stimulated genes (ISGs) are among the genes most highly induced by cytosolic *Lm* in macrophages or in *Lm*-infected mice [19], [46], [47].

Typically, bacteria activate type I IFN signaling through TLR-dependent mechanisms, which involve recognition of LPS from Gram-negative organisms, or via TLR-independent cytosolic receptors that respond to bacterial nucleic acids either endocytosed or secreted directly into host cells [48], [49]. *Lm* activates cell surface receptor and cytosolic receptor signaling pathways [50]. Specifically, cytosolic bacteria trigger a unique cytokine response that includes production of type I IFNs [11], [14], [51].

Although several cytokines are important for immunity to *Lm* infection, type I IFNs appear to be deleterious to the host [51], [52], [53], [54] however, opposing roles have been described [55]. Whereas type I IFNs provide protection against viruses, their role in bacterial infection is less clear [24], [56]. In addition, the diverse effects of type I IFNs include increasing sensitivity to *Lm*-induced cell death [57]; inducing the downregulation of the IFNγ receptor gene (IFNGR) and thus rendering the host cells refractory to IFNγ, which is crucial to achieving host resistance to *Lm* [58]; and regulating the expression of chemokines important for leukocyte recruitment [55], [59], [60]. And indeed, monocytes recruitment to Lm infected spleen is maintained in the absence of either MyD88 or IFNAR signaling. However, deficiency of both pathways impair cell migration and increased rather than decreased susceptibility to infection indicating that type I IFN has both positive and negative effects on the resistance to Lm infection [55].

In this study, we confirmed that IFNβ gene expression is strongly induced in DCs in response to Lm infection, and that the peak of this production (24 hr p.i.) is consistent with that described previously [61]. Our data indicated that type I IFNs are not induced by Lm with the same early kinetics observed *in vi*rus infections, which cause these cytokines to be produced within a few hours after infection.

This observation is compatible with the cytosolic detection of *Lm* bacteria by an as yet unknown intracellular receptor. We postulated that intracellular recognition as opposed to recognition by a cell surface receptor could lead to specific pathogen adaptations.

Thus, we sought to determine whether the delay in IFNβ production could provide a selective advantage for *Lm* spreading during the very early phase of an activated innate immune response. We hypothesized that the timing of IFNβ production could alter the nascent host innate response, and for this reason, we studied the effect of the lack of IFNβ production at 5 hr p.i. To determine the impact of delayed IFNβ production in *Lm* infection, we first performed a survival assay in which the mice were infected with *Lm* with or without the administration of exogenous IFNβ. We demonstrated that at the very early phase of *Lm* infection, treatment with IFNβ as early as 1 hr after bacteria inoculation allowed the mice to survive the infection (Figure 6). These results suggest that delayed IFNβ production early during the initial phase of *Lm* infection may provide a selective advantage for the bacterium because the innate immune system is not properly activated and/or regulated. Our findings are consistent with those of previous studies showing that cytokine delays indeed affect the outcome of both innate and adaptive immune responses [62], [63], [64]. We propose that the presence of an early source of exogenous IFNβ exert an important immuno-regulatory role in that it enhances cellular recruitment (Tip-DC), activation/regulation (NK cells) whereas inhibits other cellular types that could cause excessive tissue damage [65], [66].

The large amount of IFNβ induced by a lethal dose of Lm at 24 h p.i. when the inflammatory responses are fully activated and the bacterial burden has probably exceeded above the threshold level for survival, the effect of IFNβ on cellular apoptosis may conceal the beneficial effect of IFNβ induction. For this reason, we believe that our data are not in contradiction with previous reports on detrimental effect of type I IFNs because we examined the regulatory role of exogenous IFNβ at 5 h p.i. At this early time point of Lm infection the inflammatory response is not yet fully activated and the positive effect of IFNβ can be appreciated. And indeed, in these conditions the mice early treated with IFNβ are able to survive lethal infection.

We also examined IFNβ induction in DCs infected *in vitro* by other Gram-positive bacteria. We found that, for example, *Lactococcus lactis* is able to induce IFNβ production with a peak at 8 hr p.i. rather than at the 24 hr p.i. time point, as occurs in *Lm*-infected DCs (Figure S5). This data suggested that IFNβ production is differentially modulated in DCs during infections by different Gram-positive bacteria.

Mechanistically, we found that *Lm*-infected DCs were not able to activate NK cells either *in vitro* or *in vivo*, both in terms of inducing IFNγ production and in stimulating the lysis of target cells, suggesting that innate immunity mediated by NK cells is unpaired at 5 hr p.i. in our model. NK cell activation could be reconstituted by adding IFNβ in the co-cultured system, suggesting that IFNβ is indeed required for early NK cell activation. Nevertheless, bacterial counts in the spleen of infected mice were only moderately reduced indicating that other mechanisms were operating in our model. Recent reports suggest that NK cells and a cell population that is CD11b+Ly6G+ exhibit tissue protective properties in the context of innate immunity, a function that could be enhanced by the presence of IFNβ [67], [68].

Our results imply that IFNβ maybe able to induce disease tolerance by inducing tissue protection instead of disease resistance by reducing bacterial burden, a mechanism that has been recently suggested by Medzhitov et al. [27]. The activation of the above mechanism should explain the observed increase in mice survival in the group of IFNβ treated animals. However, the exact mechanisms remain unknown and it is the focus of our ongoing studies.

In conclusion, the innate immune response is a complex interplay between cells and soluble factors that, in the right context and environment, together provide a framework to combat infection. However, successful pathogens have evolved mechanisms that subvert these ancient controls, allowing them to counteract the innate immune responses of their hosts.

Here we suggest that a transient impaired of NK cell activation/regulation may be an additional strategy employed by *Lm* to avoid innate immune activation and that IFNβ can regulate antibacterial activity in the very early phases of acute infection acting as a positive regulatory molecule.

## Materials and Methods

### Ethics Statement

All animal experiments were performed using protocols approved by University of Milano-Bicocca Animal Care and Use Committee. All experimental procedures were carried out in strict accordance with the 2003/65/CEE European directive for animal experimentation. Protocols used in this study were approved by the Italian Ministry of health under the protocol number 3–2001. Mice were housed in containment facilities of the animal facility and maintained on a regular 12∶12 hour light:dark cycle with food and water ad libitum.

### Mice and Reagents

All mice were bred on a C57BL/6 background and animals were housed under pathogen-free conditions. C57BL/6 mice were purchased from Charles River and were maintained in our animal facility at the University of Milano-Bicocca.

Monoclonal antibodies for FACS analysis, cell purification and intracellular staining were purchased from Becton and Dickinson (Franklin Lakes, NJ, USA). Recombinant mouse IFNβ and IFNα and the IFNβ mouse ELISA kit were obtained from PBL Medical Laboratories (Piscataway, NJ, USA). The BD OptEIA mouse IFNγ ELISA kit was used to test IFNγ in co-culture supernatants. YAC-1 cells were purchased from ATCC (American Type Culture Collection, Rockville, MD).

### Microarray Assay and Analysis

We harvested 10^7^ D1 cells in the immature state or after 4 h, 8 h, 12 h or 24 h of stimulation. Total RNA was isolated with Trizol Reagent (Invitrogen, Life Technologies, Karlruhe, Germany) and purified on a Qiagen RNeasy column (Qiagen, Hilden, Germany) to remove small fragments. RNA quality was assessed on an Agilent 2100 Bioanalyzer RNA 6000 Nano LabChip (Agilent Technologies, Palo Alto, CA). Only samples with intact total RNA profiles (retention of both ribosomal bands and the broad central peak of mRNA) were used for the microarray and quantitative RT-PCR gene expression analyses. In vitro transcription (IVT) products were generated and oligonucleotide array hybridization and scanning were carried out according to the instructions supplied by Affymetrix (Santa Clara, CA). We used 10 to 16 mg of total RNA from each sample and T7-linked oligo-dT primers for first-strand cDNA synthesis. The fragmented biotinylated cDNA (15 mg) was hybridized onto the MOE430A GeneChip (Affymetrix), using the recommended procedures for prehybridization, hybridization, washing and staining with streptavidin–phycoerythrin (SAPE). Array images were analyzed with the RMA algorithm [69]. Samples displaying a signal ratio >3.0 for the b-actin and GAPDH probe sets were considered to be poor-quality targets and were excluded from the dataset. Genespring (Silicon Genetics) microarray analysis software was used for further analysis.

Microarray data were deposited in the ArrayExpress database (E-MEXP-3159 and E-MEXP-3158) and followed MIAME requirements.

### RNA Preparation and PCR

RNA was isolated from tissue or cells using TRIzol reagents (Invitrogen) and extracted on an RNeasy mini kit column (RNA isolated from the spleen) or micro kit column (RNA isolated from purified cells) (Qiagen, Valencia, CA). RNA was then quantified and used in a reverse transcriptase (RT) reaction using the high capacity cDNA reverse transcription kit (Applied Biosystem). cDNA was used for PCR amplification.

Relative levels of RNA were determined by quantitative PCR on a real time detection system (7500 real time PCR system, Applied Biosystems) using a TaqMan gene expression assay for IFNβ. Gene-specific transcript levels were normalized to PPIA mRNA. Amplification conditions were as follows: 50°C for 2 min, 95°C for 10 min, 40 cycles of 95°C for 15 sec, and 60°C for 1 min.

### Bacteria

*Listeria monocytogenes (Lm)* EGD pNF8 (BUG) was used for infection studies. This strain is *Lm* EGD strain (BUG600) carrying the pNF8 plasmid, which contains the gene encoding the GFP protein [70]. The replicative plasmid was maintained in the strains by growing them on brain heart infusion (BHI) agar containing 5 mg/ml of erythromycin. *Listeria innocua* (*Li*) and *Lm* were provided by the P. Cossard’s laboratory. *Escherichia coli* (*E. coli*) DH5a strain (Invitrogen). Bacteria were grown in brain heart infusion broth or LB (Sigma Aldrich, St Louis, MO, USA) to a mid-logarithmic phase and stored in small aliquots of 10% glycerol stocks at –80°C until use. *Lm* concentration was quantified by plating serial dilutions on BHI agar plates containing 5 mg/ml of erythromycin and counting colonies after growth at 37°C for 24–36 hours.

### Infections with Bacteria

For *in vitro* infections with *Lm,* bacteria were thawed from glycerol stocks, washed in PBS, diluted into appropriate media and added to cells at a multiplicity of infection (MOI) of 20 or 40, as indicated. The Lm MOI used kept cell mortality between 10–20%. Bacterial numbers were confirmed by plating serial dilutions onto BHI agar plates containing 5 mg/ml of erythromycin. Infection was allowed to proceed for 1 h at which time extracellular bacteria were washed away, and gentamicin-containing medium (final concentration 50 µg/ml) was added to prevent extracellular bacterial growth. After another 60 min, medium was changed to medium containing 10 µg/ml gentamicin.

For intravenous infection of *Lm,* bacteria were thawed from glycerol stocks, washed and diluted in PBS before injection (200 ml) into the lateral vein of the tail. The infection dose was checked by plating serial dilutions of agar plates containing 5 mg/ml of erythromycin and counting colonies after growth at 37°C for 24–36 hours. Mice were injected with sub-lethal doses (5×10^4^−10^5^ CFU) or lethal doses (10^6^ CFU). *Escherichia coli (E. coli)* was grown on LB and added to cells at a MOI of 20. Mice were injected with 10^7^ *E. coli*. For determination of bacterial load, mice were killed 5 hours post infection, and spleens were homogenized in 0.2 % Triton-X100 solution. Serial dilutions of homogenates were plated on BHI agar plates containing 5 mg/ml of erythromycin, and colonies counted after growth at 37°C for 24–36 hours.

### Preparation of BMDCs

Bone marrow cells from C57BL/6 mice were cultured in IMDM (Euroclone, Milan, Italy) supplemented with 2 mM L-glutamine, 100U/ml penicillin, 100 mg/ml streptomycin, 50 mM 2-mercaptoethnol (all from Sigma, St Louis, MO, USA), 10% heat-inactivated FBS (IMDM complete medium) and 10% supernatant of granulocyte-macrophage colony-stimulating factor (GM-CSF)-transduced B16 tumor cells [71]. Fresh medium was added every three days. After 7–10 days of culture, cells were analyzed for CD11c expression; cultures with 75–80% CD11c-positive cells were used in assays.

### NK Cell Purification

NK cells were purified from C57BL/6 mice by positive selection from splenocytes. After lysis of red blood cells, splenocytes were stained with biotinylated anti-pan-NK cell (DX5) antibody (10 mg/ml), washed and incubated with streptavidin Microbeads (Miltenyi Biotech, Bergish Gladbach, Germany). Cells were positively selected using MS columns, according to the manufacturer’s recommendations. Resultant NK cell populations were used if at least 80% were NK1.1 positive.

### Purification of Splenic Dendritic Cells

Dendritic cells were purified from C57BL/6 mice by positive selection from splenocytes. Spleens were treated with collagenase before purification. After red blood cell lysis, splenocytes were incubated with CD11c^+^ Microbeads (Miltenyi Biotec.) and positively selected using MS columns, according to the manufacturer’s recommendations. Purified cells were stained with CD11c-PE and anti-MHC class II-PE to check the efficiency of purification. Purified cells were usually 90% CD11c^+^ and 95% MHC class II. Both positive (CD11c^+^) and negative fractions (CD11c^−^) were used for PCR studies.

### NK-DCs Co-cultures

Co-culture experiments were performed with NK cells derived from C57BL/6 mice. Wild-type BMDCs were resuspended in IMDM complete medium containing 10% GM-CSF supernatant without antibiotics and plated in 96-well plates (10^5^ cells/well). DCs were treated with *Lm* or *E.coli* DH5a with an MOI of 20 for 1 hour and then supplemented with IMDM complete medium containing 10% GM-CSF supernatant with gentamycin (50 mg/ml), penicillin (100 U/ml), and streptomycin (100 mg/ml). Activated BMDCs were cultured with rIFNβ where indicated (100 U/ml). NK cells were added (10^5^ cells/well) directly to the culture after 30 minutes and supernatants were tested for IFNγ production 18 hours later.

### *In vivo* Activation of NK Cells

Wild-type mice were injected intravenously (iv) with 10^6^ *Lm* with or without subsequent injection of IFNβ (38000U). Spleens were removed after 5 hours and analyzed for NK cell activation. For intracellular staining, single cell suspensions were prepared and incubated with brefeldin A (10 µg/ml, Sigma), ionomycin (100 ng/ml, Sigma), and phorbol 12-myristate 13-acetate (50 ng/ml, Sigma) for 3 hours. Cells were then stained with FITC-labeled anti-IFNγ monoclonal antibody. NK cells were identified by staining with PE-labeled monoclonal anti-CD49b (clone HMa2) antibodies. Intracellular staining for IFNγ was performed using the BD kit according to the manufacturer recommendations (Becton Dickinson, San Diego, CA, USA). Cells were then analyzed on a FACScan (Becton Dickinson, San Diego, CA, USA). The ability of NK cells to kill YAC-1 target cells was evaluated in a DELFIA cytotoxicity assay, as described below. Mice were injected iv with 10^6^ Lm with or without subsequent injection of IFNβ (38000U). Spleens were removed after 5 hours and splenocytes were co-cultured with YAC-1 cells. Cytotoxicity was measured after three hours using a time-resolved fluorometer (Victor, Perkin Elmer).

### Cytotoxicity Assay

YAC-1 cells were grown in IMDM (Euroclone, Milan, Italy) medium, supplemented with 2 mM L-glutamine, 100 U/ml penicillin, 100 mg/ml streptomycin, 50 mM 2-mercaptoethnol (all from Sigma, St Louis, MO, USA), 10% heat-inactivated FBS (IMDM complete medium), until the day of the experiment. On the day of the experiment, cells were loaded with Delfia bis (acetoxymethyl)2,2′:6′,2″-terpyridine-6,6″-dicarboxylate (BADTA) for 30 minutes at 37°C, in 5% CO_2_. Splenocytes from treated mice were incubated with 2×10^4^ BADTA-labeled cells in a 96-well microplate for three hours (to obtain a target:NK cell ratio of approximately 1∶5). After incubation, released BADTA was measured in Europium solution (Perkin Elmer) by time-resolved fluorescence using a 1420 multilabel counter Victor^3^ plate reader. Percent of specific lysis was determined as (mean experimental release-mean spontaneous release)/(mean maximum release-mean spontaneous release)X100.

### Statistical Analysis

All experiments were repeated at least three times. Asterisks(*) in the Figures indicate differences deemed significant (P<0.01) by a two-tailed Student’s T test. All error bars in graphs indicate SE for three samples per experimental group.

## Supporting Information

Figure S1 **Flow cytometry analysis of DC maturation marker expression during bacterial challenge.** D1 cells (A) or BMDCs (B, C) were activated with the stimuli indicated. Different colors represent different MOI values. A: green 1∶20, pink 1∶40, light blue 1∶80; B: green 1∶20, pink 1∶40, light blue 1∶80; C: pink 1∶20. Untreated D1 cells or BMDCs are shown in black.
(TIF)

Figure S2 **Functional annotation of differentially expressed genes using KEGG.** Significant functional annotation of KEGG for pathway enrichments. A list of KEEG pathways induced in DCs by *Lm* (A) and by *Li* (B) are shown. The pathways marked in red are those that are the most statistically significant. The blue circles indicate genes modulated positively and negatively in the specific pathway analyzed. Also listed in the Figure are genes involved in the cytokine-cytokine receptor interactions induced in the DCs by the two bacteria strains.
(TIF)

Figure S3 **Functional annotation of differentially expressed genes using gene ontology (GO) annotation.** DEGs in the 2–4 hr p.i. interval were defined as early responsive genes and annotated for *Lm* (A) and *Li* (B) infection-related DEGs using the GO for functional enrichment. The Figure lists the most significant enrichments obtained and includes lists of the genes included in the functional classes. *Lm*-related DEGs are most enriched in the GO biological processes of “locomotion" and “chemotaxis" whereas the DEGs related to *Li* infection are enriched in the processes of “immune system process" and “immune response".
(TIF)

Figure S4 **GO functional annotation of differentially expressed genes induced specifically by** ***Li*** **infection.** Specific DEGs induced by *Li* in DCs are functional annotated by GO biological process. The relevant genes that are significantly enriched in the “phosphate metabolic process" and in the “regulation of apoptosis" are listed.
(TIF)

Figure S5 **IFNβ production by** ***Lactococcus lactis*** **infection.** An IFNβ gene sequence (400 bp) was amplified by RT-PCR. D1 cells were infected with *Lm* (MOI of 70) and *L. lactis* (MOI of 1,000) for the times indicated. The β-actin gene was used as normalization control. Data shown is representative of three independent experiments.
(TIF)
