# Supplementary figures and images for: The Timing of IFNβ Production Affects Early Innate Responses to Listeria monocytogenes and Determines the Overall Outcome of Lethal Infection
Source: PLoS One. 2012 Aug 17;7(8):e43455. doi: 10.1371/journal.pone.0043455 (PMC3422257; doi:10.1371/journal.pone.0043455)

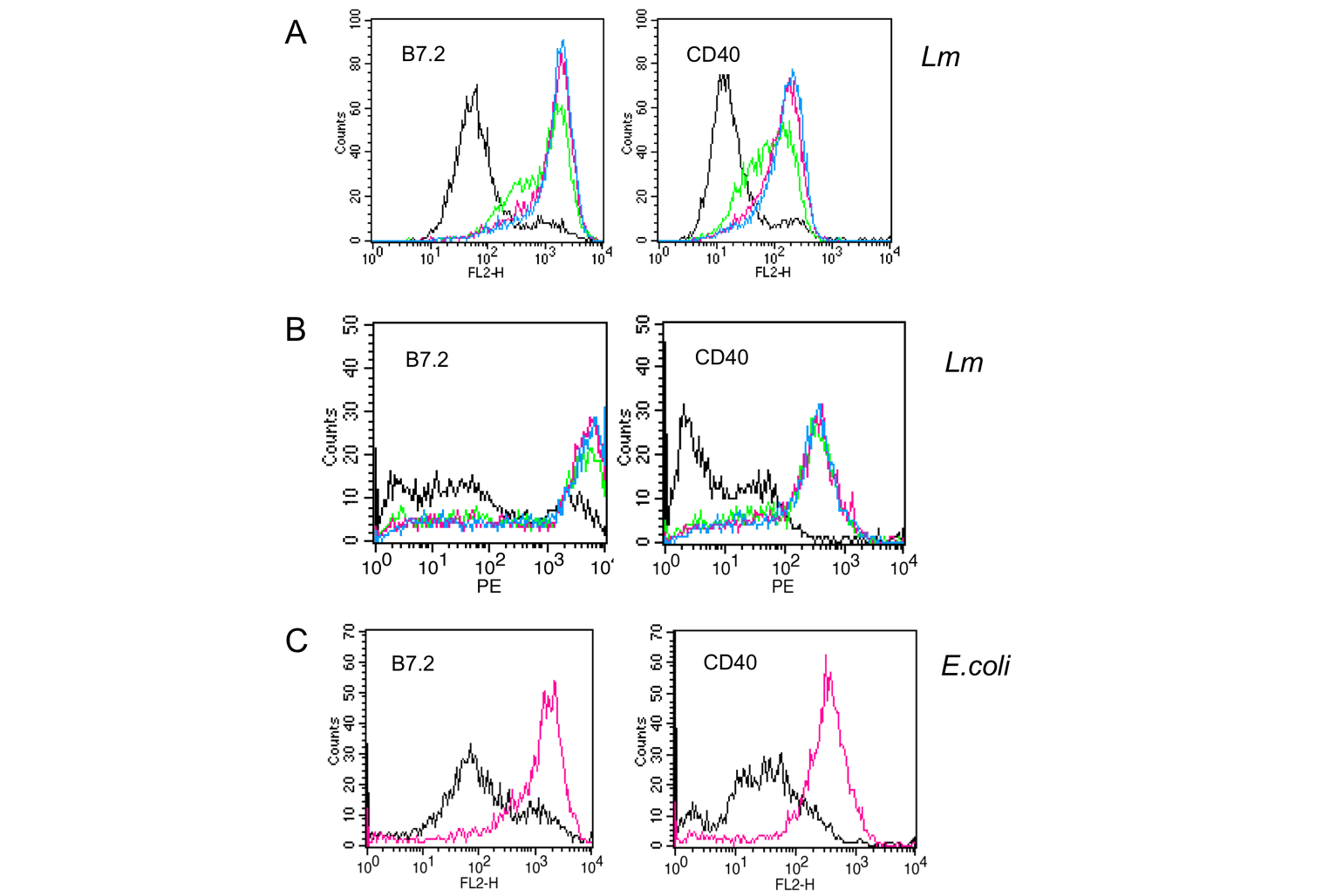

Supplement: Figure S1 — Flow cytometry analysis of DC maturation marker expression during bacterial challenge. D1 cells (A) or BMDCs (B, C) were activated with the stimuli indicated. Different colors represent different MOI values. A: green 1∶20, pink 1∶40, light blue 1∶80; B: green 1∶20, pink 1∶40, light blue 1∶80; C: pink 1∶20. Untreated D1 cells or BMDCs are shown in black. (TIF) [file pone.0043455.s001.tif]

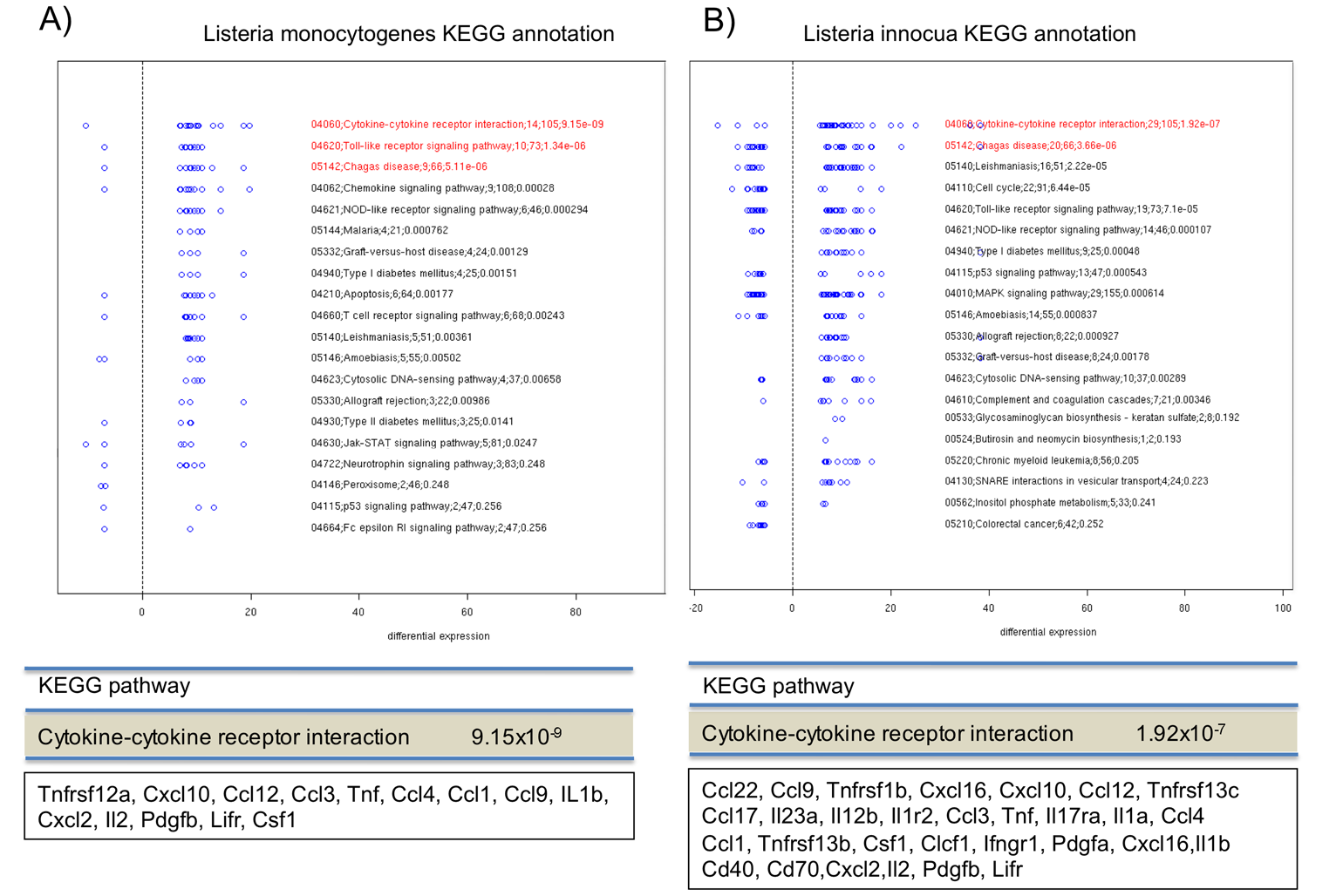

Supplement: Figure S2 — Functional annotation of differentially expressed genes using KEGG. Significant functional annotation of KEGG for pathway enrichments. A list of KEEG pathways induced in DCs by Lm (A) and by Li (B) are shown. The pathways marked in red are those that are the most statistically significant. The blue circles indicate genes modulated positively and negatively in the specific pathway analyzed. Also listed in the Figure are genes involved in the cytokine-cytokine receptor interactions induced in the DCs by the two bacteria strains. (TIF) [file pone.0043455.s002.tif]

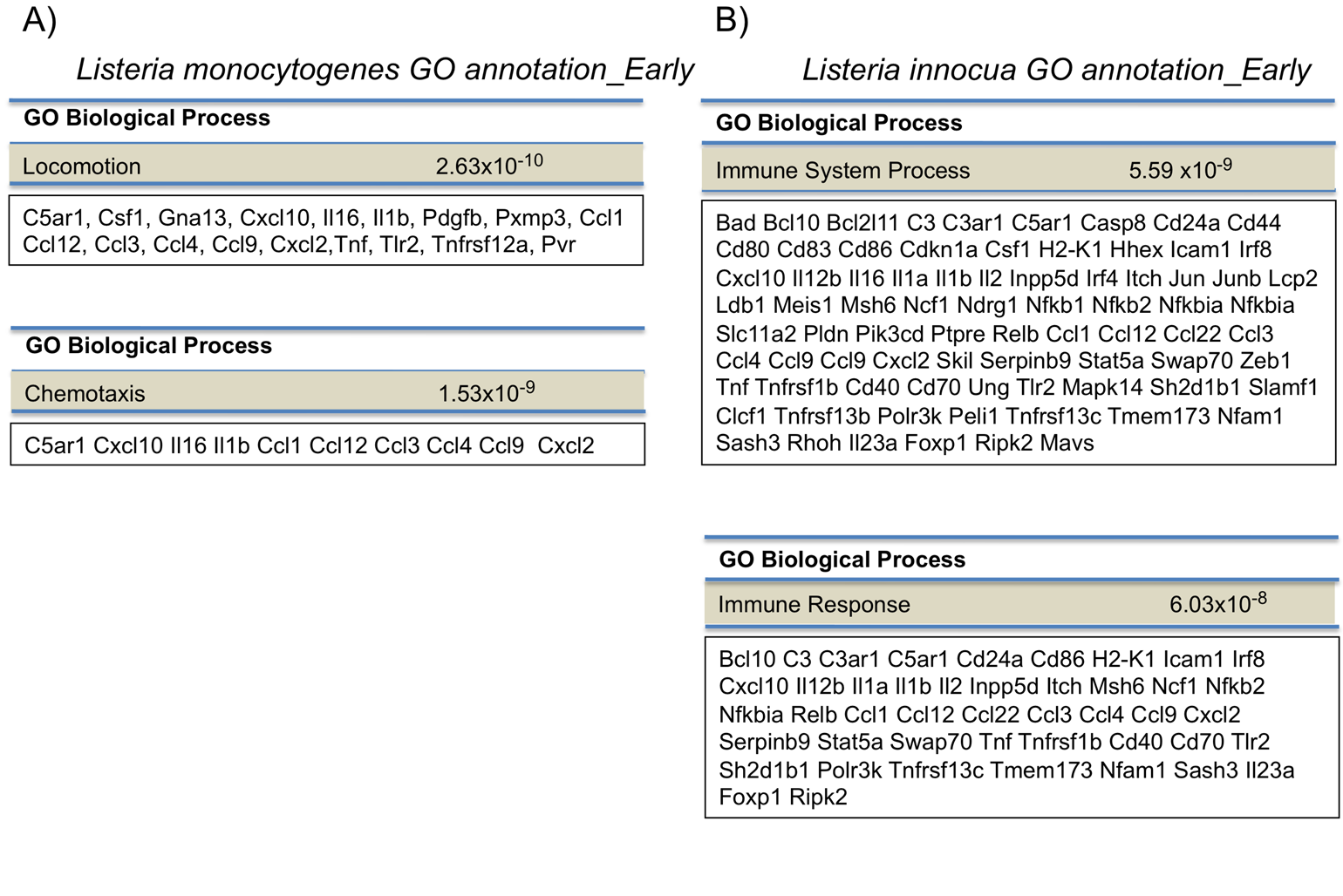

Supplement: Figure S3 — Functional annotation of differentially expressed genes using gene ontology (GO) annotation. DEGs in the 2–4 hr p.i. interval were defined as early responsive genes and annotated for Lm (A) and Li (B) infection-related DEGs using the GO for functional enrichment. The Figure lists the most significant enrichments obtained and includes lists of the genes included in the functional classes. Lm-related DEGs are most enriched in the GO biological processes of “locomotion" and “chemotaxis" whereas the DEGs related to Li infection are enriched in the processes of “immune system process" and “immune response". (TIF) [file pone.0043455.s003.tif]

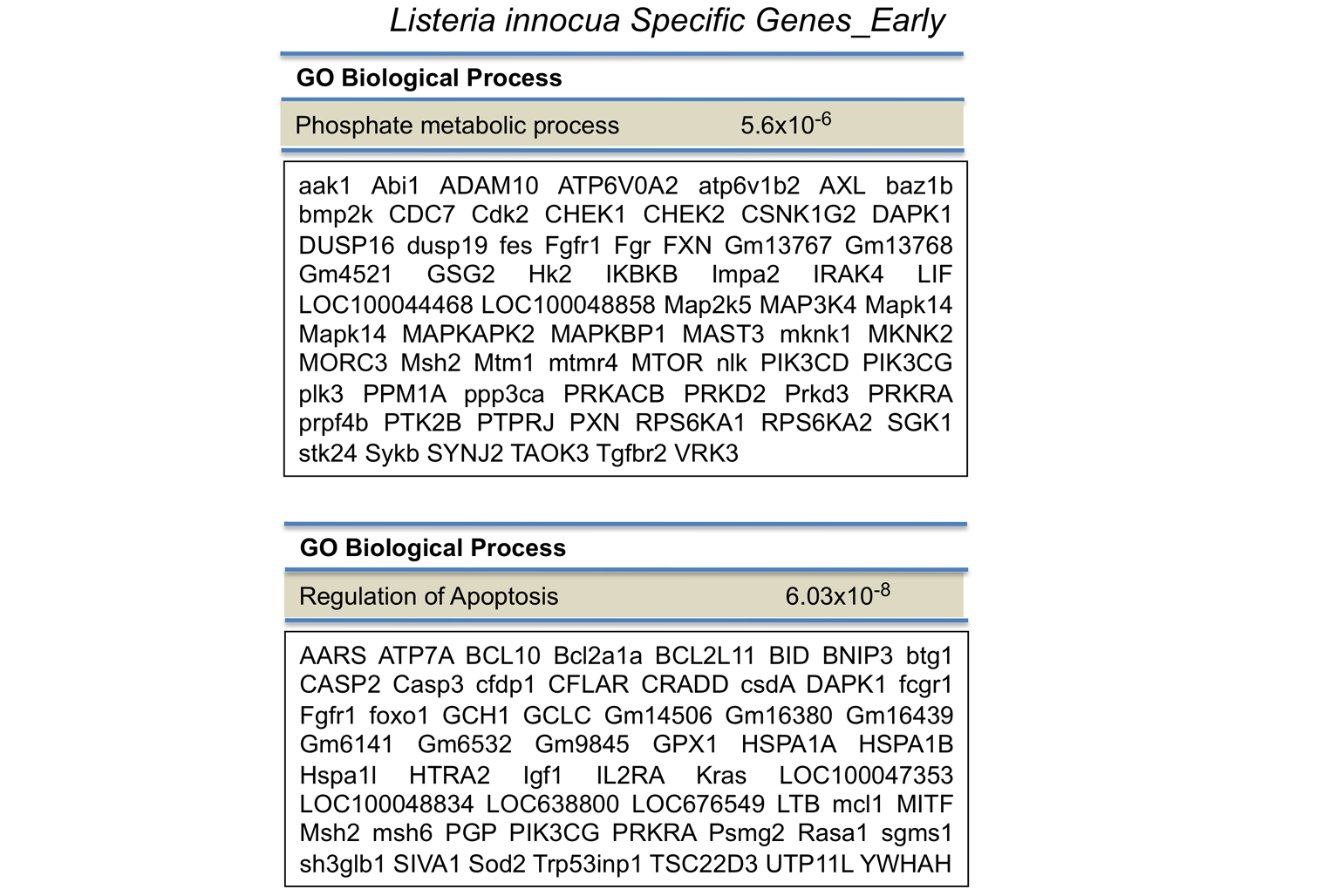

Supplement: Figure S4 — GO functional annotation of differentially expressed genes induced specifically by Li infection. Specific DEGs induced by Li in DCs are functional annotated by GO biological process. The relevant genes that are significantly enriched in the “phosphate metabolic process" and in the “regulation of apoptosis" are listed. (TIF) [file pone.0043455.s004.tif]

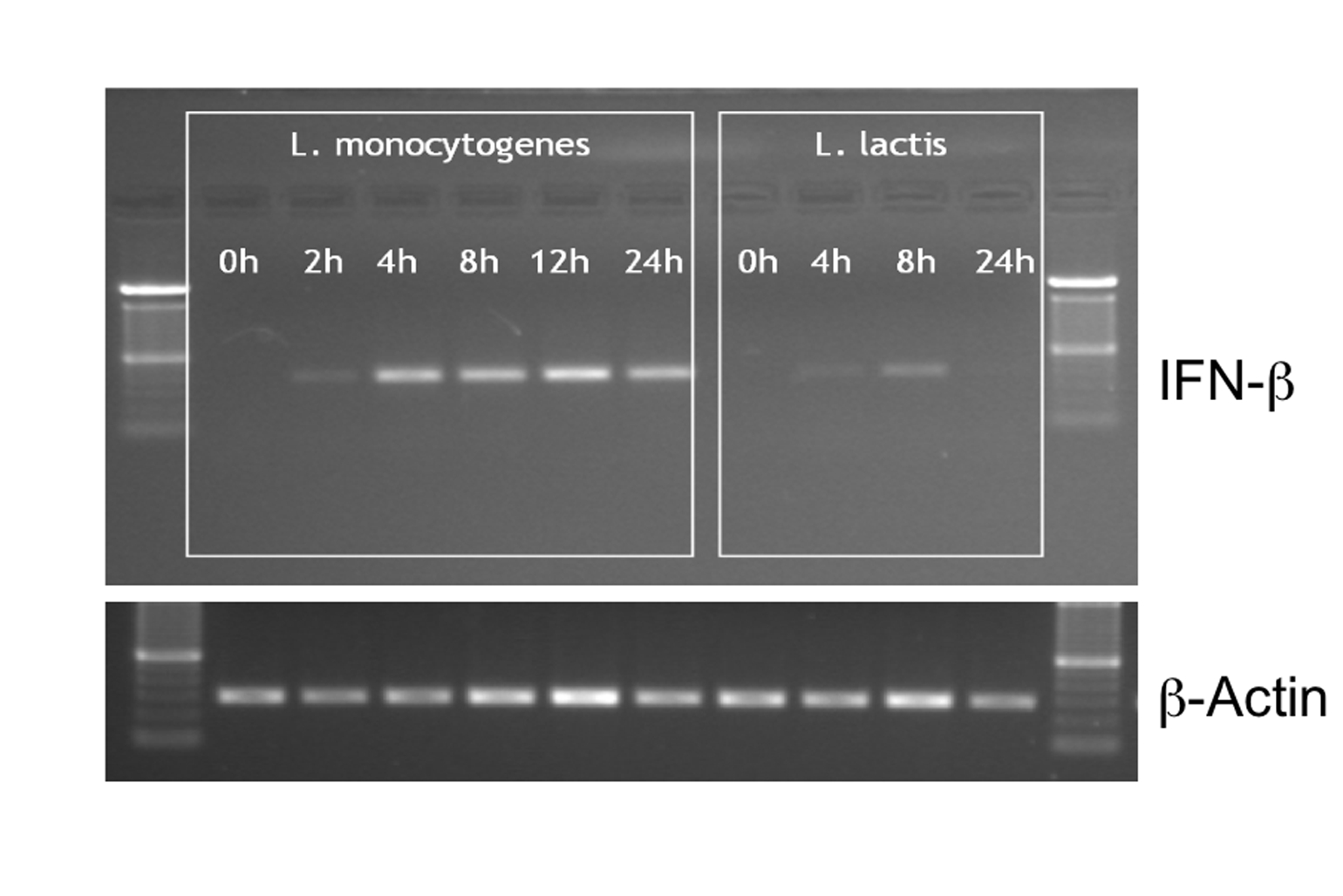

Supplement: Figure S5 — IFNβ production by Lactococcus lactis infection. An IFNβ gene sequence (400 bp) was amplified by RT-PCR. D1 cells were infected with Lm (MOI of 70) and L. lactis (MOI of 1,000) for the times indicated. The β-actin gene was used as normalization control. Data shown is representative of three independent experiments. (TIF) [file pone.0043455.s005.tif]
